
JOURNAL INFORMATION
==============================
NLM Title Abbreviation: ScientificWorldJournal
Journal ID: ScientificWorldJournal
NLM Journal ID: 101131163
Journal ID: TSWJ

The Scientific World Journal

ISSN: 2356-6140
EISSN: 1537-744X
Publisher: Wiley

ARTICLE INFORMATION
==============================
PMCID: PMC5763820
PMID: 20694453
DOI: 10.1100/tsw.2010.157
Article ID: 184947
Article version: 1
Subjects: Editorial

Resolution of Acute Inflammation and the Role of Lipid Mediators

Clària Joan *
Department of Biochemistry and Molecular Genetics, Hospital Clínic, IDIBAPS, CIBEK, and CIBERehd, University of Barcelona, Spain
*Joan Clària: jclaria@clinic.ub.esAcademic Editor: Claire Redhead

Publication date: 2010
Electronic publication date: 2010 Aug 3
Volume: 10
First page: 1553
Last page: 1555
Received 2010 Jul 13; Accepted 2010 Jul 13
Copyright: Copyright © 2010 Joan Clària.
Copyright year: 2010
License: This is an open access article distributed under the Creative Commons Attribution License, which permits unrestricted use, distribution, and reproduction in any medium, provided the original work is properly cited.
License URL: https://creativecommons.org/licenses/by/3.0/

==============================
Editorial
Special Issue: Resolution of Acute Inflammation and the Role of Lipid Mediators
*TheScientificWorldJOURNAL* (2010) 10, 1553–1555
ISSN 1537-744X; DOI 10.1100/tsw.2010.157

TheScientificWorldJOURNAL
www.thescientificworld.com

### Resolution of Acute Inflammation and the Role of Lipid Mediators

Joan Clària

*Department of Biochemistry and Molecular Genetics, Hospital Clínic, IDIBAPS, CIBEK, and CIBERehd, University of Barcelona, Spain*

**E-mail**: jclaria@clinic.ub.es

*Received July 13, 2010; Accepted July 13, 2010; Published August 3, 2010*

---

Bioactive lipid mediators originating from the cleavage of structural lipid components of cellular membranes represent one of the most potent classes of endogenous inflammatory mediators. Eicosanoids, which are a large family of compounds generated from arachidonic acid, are a paradigmatic example of this class of lipid mediators. Arachidonic acid is an essential ω-6-polyunsaturated fatty acid (PUFA) primarily found esterified in the 2-acyl position of phospholipids in all mammalian outer- and intracellular membranes. Upon activation of phospholipase $A_2$, arachidonic acid is released from membrane phospholipids, and its free acid becomes available as a substrate for the intracellular biosynthesis of eicosanoids through two major enzymatic routes: the cyclooxygenase (COX) and lipoxygenase (LO) pathways. The COX pathway results in the formation of prostaglandins (PGs) and thromboxane $A_2$, which are known for their powerful physiological properties and PGs specially, for their critical role in the inflammatory response. On the other hand, the LO pathway comprises three major LOs: 5-, 12-, and 15-LO, with 5-LO, which converts arachidonic acid into 5(S)-hydroxyeicosatetraenoic acid and leukotrienes (LTs), a consolidated pharmacological target in inflammation.

Since prolonged inflammation is detrimental to the host, higher organisms have evolved protective mechanisms to ensure resolution of the inflammatory response in a limited and specific time and space manner. Resolution of inflammation is, at present, envisioned not as a mere passive process of dilution of inflammation, but a highly orchestrated and complex process in which many endogenous anti-inflammatory and proresolving mediators counteract the effects of proinflammatory mediators. Although the mediators and mechanisms implicated in the resolution of inflammation have remained largely ignored, at present, it is quite clear that the same lipid mediators that initially trigger the inflammatory response also signal the termination of inflammation by stimulating the biosynthesis of anti-inflammatory and proresolving lipid autacoids. For instance, both $PGE_2$ and $PGD_2$ transcriptionally activate the expression of 15-LO in human neutrophils (PMN), switching the mediator profile of these cells from the proinflammatory $LTB_4$ to the anti-inflammatory compound lipoxin $A_4$. This class switch in eicosanoid production and phenotypic change in mediator profiles generated from arachidonic acid in resolving tissues provide a temporal and spatial dissociation of eicosanoid biosynthesis that is emerging as a critical factor in the resolution of inflammation. Nowadays, the lipid mediators most recognized as “stop signals” of inflammation are (1) the lipoxins, which were the first-identified ω-6-PUFA–derived lipid mediators with potent immunomodulatory and anti-inflammatory properties; (2) the recently described ω-3-PUFA–derived mediators resolvins and protectins; and (3) the cyclopentenone PGs of the D series. Interestingly, these families of endogenous proresolution molecules are not immunosuppressive, but instead stimulate and accelerate resolution of inflammation by activating specific mechanisms to restore tissue homeostasis. These anti-inflammatory and proresolving mediators exert a strict control of the resolution

©2010 with author.
Published by TheScientificWorld; www.thescientificworld.com process, and not only stop PMN and eosinophil functions, but also pave the way for monocyte migration and their differentiation to phagocytosing macrophages, which remove dead cells and then terminate the inflammatory response. A concept that is currently of interest is that loss or deterioration of tissue function during chronic inflammation is the result of an inappropriate inflammatory response that remains uncontrolled because of the lack of the intrinsic capacity of the tissue for complete resolution. Therefore, the modulation of these "stop signals" that promote the timely resolution of inflammation is emerging as a strategy to maintain inflammation self-limiting and to prevent tissue injury and disease.

This special issue on "Resolution of Acute Inflammation and the Role of Lipid Mediators" is devoted to describe the current status, characteristics, and progress in this class of "stop signals", especially on the role of lipoxins and the recently described ω-3-PUFA–derived lipid mediators, resolvins and protectins, in the resolution of inflammation. The paper by Mario Romano[1] reviews the available literature on lipoxins and aspirin-triggered lipoxins, which are eicosanoids emerging as pivotal break signals of inflammation and promoters of resolution. Their biosynthetic pathways, bioactions, and involvement in human disease are discussed as well as their potential use as a new class of anti-inflammatory drugs. The contribution by Gerard Bannenberg[2] discusses different aspects of the therapeutic applicability of endogenous anti-inflammatory and proresolving lipid mediators, and indicates that the development of innovative pharmacotherapy based on these endogenous compounds presents novel prospects for the treatment of inflammatory diseases. In their review, Arita and collaborators[3] focus on the biosynthesis, chemical structure, and action of resolvins in immune systems and describe the identification of these novel lipid mediators derived from ω-3-PUFA as potent immune regulators with anti-inflammatory and/or proresolving properties. The contribution by Carlo and Levy[4] describes the resolution programs that prevent bystander lung injury from excessive inflammation. These authors review rapidly emerging information on cellular and molecular mechanisms for lipoxins, resolvins, and protectins as potent anti-inflammatory and proresolving actions in the airway. González-Périz and Clària[5] review the switch to the formation of anti-inflammatory and proresolving ω-3-PUFA–derived lipid autacoids, such as resolvins and protectins, during the resolution of adipose tissue inflammation. These authors recognize the role of the endogenous compounds in stopping the state of chronic "low-grade" inflammation in adipose tissue, which predisposes to obesity-related comorbidities, such as insulin resistance and nonalcoholic fatty liver disease. The contribution of Gronert and Liclian[6] summarizes the current state of the field regarding the protective endogenous lipid circuits that have emerged as key components of endogenous pathways that preserve ocular function and limit the sequelae of ocular injury. Furthermore, the review by Börgeson and Godson[7] focuses on the evidence for molecular circuits of resolution mediated by lipid mediators, such as lipoxins, resolvins, and protectins, in renal disease. Finally, Díez-Dacal and Pérez-Sala[8] elaborate on electrophilic prostanoids like the cyclopentenone PGs acting through signaling mechanisms dependent on cysteine modification. These lipid mediators unveil a critical interplay between the cellular redox status, the electrophilic/antioxidant response, and the resolution of inflammation, which is already yielding novel anti-inflammatory targets.

### REFERENCES

1. Romano, M. (2010) Lipoxin and aspirin-triggered lipoxins. *TheScientificWorldJOURNAL* **10,** 1048–1064.
2. Bannenberg, G.L. (2010) Therapeutic applicability of anti-inflammatory and proresolving polyunsaturated fatty acid–derived lipid mediators. *TheScientificWorldJOURNAL* **10,** 676–712.
3. Seki, H., Sasaki, T., Ueda, T., and Arita, M. (2010) Resolvins as regulators of the immune system. *TheScientificWorldJOURNAL* **10,** 818–831.
4. Carlo, T. and Levy, B.D. (2010) Molecular circuits of resolution in airway inflammation**.** *TheScientificWorldJOURNAL* **10,** 1386–1399.
5. González-Périz, A. and Clària, J. (2010) Resolution of adipose tissue inflammation. *TheScientificWorldJOURNAL* **10,** 832–856.
6. Gronert, K. and Liclian, E.L. (2010) Molecular circuits of resolution in the eye. *TheScientificWorldJOURNAL* **10,** 1029–1047.
7. Börgeson, E. and Godson, C. (2010) Molecular circuits of resolution in renal disease. *TheScientificWorldJOURNAL*

**10,** 1370–1385.
8. Díez-Dacal, B. and Pérez-Sala, D. (2010) Anti-inflammatory prostanoids: focus on the interactions between electrophile signaling and resolution of inflammation. *TheScientificWorldJOURNAL* **10,** 655–675.

**This article should be cited as follows**:

Clària, J. (2010) Resolution of acute inflammation and the role of lipid mediators. *TheScientificWorldJOURNAL* **10,** 1553–1555. DOI 10.1100/tsw.2010.157.
